# Enhanced Attentional Bias towards Sexually Explicit Cues in Individuals with and without Compulsive Sexual Behaviours

**DOI:** 10.1371/journal.pone.0105476

**Published:** 2014-08-25

**Authors:** Daisy J. Mechelmans, Michael Irvine, Paula Banca, Laura Porter, Simon Mitchell, Tom B. Mole, Tatyana R. Lapa, Neil A. Harrison, Marc N. Potenza, Valerie Voon

**Affiliations:** 1 Department of Psychiatry, Addenbrooke's Hospital, University of Cambridge, Cambridge, United Kingdom; 2 Behavioural and Clinical Neurosciences Institute, University of Cambridge, Cambridge, United Kingdom; 3 Cambridgeshire and Peterborough Foundation Trust, Cambridge, United Kingdom; 5 Department of Psychiatry, Brighton and Sussex Medical School, Brighton, United Kingdom; 5 Departments of Psychiatry, Neurobiology and Child Study Center, Yale University, New Haven, Connecticut, United States of America; University of Verona, Italy

## Abstract

Compulsive sexual behaviour (CSB) is relatively common and has been associated with significant distress and psychosocial impairments. CSB has been conceptualized as either an impulse control disorder or a non-substance ‘behavioural’ addiction. Substance use disorders are commonly associated with attentional biases to drug cues which are believed to reflect processes of incentive salience. Here we assess male CSB subjects compared to age-matched male healthy controls using a dot probe task to assess attentional bias to sexually explicit cues. We show that compared to healthy volunteers, CSB subjects have enhanced attentional bias to explicit cues but not neutral cues particularly for early stimuli latency. Our findings suggest enhanced attentional bias to explicit cues possibly related to an early orienting attentional response. This finding dovetails with our recent observation that sexually explicit videos were associated with greater activity in a neural network similar to that observed in drug-cue-reactivity studies. Greater desire or wanting rather than liking was further associated with activity in this neural network. These studies together provide support for an incentive motivation theory of addiction underlying the aberrant response towards sexual cues in CSB.

## Introduction

Compulsive sexual behaviour (CSB), also termed hypersexual disorder or sexual addiction, is relatively common and associated with significant distress and psychosocial impairments [1]. The frequency of CSB has been estimated to range from 2% to 4% in community and college-based young adults, with similar estimates in psychiatric inpatients [2]–[4]. CSB has been conceptualized as an impulse control disorder or a non-substance or “behavioural” addiction [5]. Based on existing data, pathological gambling (or gambling disorder) was recently reclassified in DSM-5 as a behavioural addiction [6]. However, although criteria for hypersexual disorder and other excessive conditions were proposed for DSM-5 [7], disorders relating to excessive engagement in Internet use, video-gaming or sex were not included in the main section of the DSM-5, in part due to limited data on the conditions [8]. Thus, further studies on CSB and how it might show similarities to or differences from substance use disorders may help with classification efforts and the development of prevention and treatment. Here we assess attentional bias towards sexual cues individuals with and without CSB, placing the findings in the context of attentional bias studies in individuals with substance use disorders.

Disorders of addiction are characterized by biases in selective attention towards drug cues [9]–[15]. Subjects with substance use disorders show information processing deficits in the presence of substance-related stimuli [16]. Attentional biases may be defined as tendencies for perceptions to be influenced by specific internal or external stimuli. One possible mechanism underlying attentional bias to drug cues in drug use disorders has been postulated to reflect incentive learning theory. Through the process of classical conditioning, with repeated pairing of cues and the drug, these drug cues develop an incentive value and acquire incentive-motivational properties. The incentive salience means the drug cues become more attractive, thus grabbing attention, eliciting generalized approach behaviours and becoming ‘wanted’ [16]–[18]. Attentional biases towards substance-related stimuli have been shown in substance use disorders for alcohol, nicotine, cannabis, opiates and cocaine (reviewed in [19], [20]–[22]). Several paradigms have been developed to measure attentional deficits including eye movement tasks, the Posner task, drug-related variants of the Stroop task and the dot probe task. Biases of attention in eye movements to substance-related cues have been shown in smokers [23] and individuals with cocaine addictions [24]. A modification of the Stroop Task, the addiction Stroop [19], evaluates attention to disorder-relevant cues by substitution of color words for arousal provoking words [25]. However, it has been suggested that the addiction Stroop task may be confounded by attempts to suppress attentional bias or slowing of cognitive processes as a consequence of craving rather than strictly attentional bias [26], [27]. Addiction Stroop tasks assess attempts to suppress or inhibit the attentional bias or prepotent responses to disorder-relevant cues and do not assess key features underlying attentional bias, such as facilitated attention or difficulties in disengagement [28], [29]. In contrast, the dot probe task [30], [31] in which the position of the dot probe or target is manipulated relative to the position of visually displayed drug cue or neutral images, allows for the assessment of facilitation and disengagement processes [29], [32]. Attentional bias measures assessed by the Stroop and dot probe task also do not correlate [28], [33] consistent with the measures focusing on differing processes such as response inhibition and attention allocation respectively. Thus, although the different tasks each assess responses to salient cues, the processes measured differ.

We compared CSB subjects and matched healthy volunteers using a dot probe task to assess attentional biases to sexually explicit cues versus control stimuli and neutral cues versus control stimuli. As the latency of the stimulus has been shown to play a role in whether subjects engage in an early orienting facilitation response or a later inhibitory response [34], [35], the responses were divided into early and late stimulus latencies. We hypothesized that similar to attentional biases observed to drug cues in individuals with addictions, individuals with CSB compared to healthy volunteers would have enhanced attentional bias or faster reaction times to sexually explicit cues compared to a neutral stimulus but not to a neutral person cue compared to a neutral stimulus for early stimulus latencies.

## Methods

### Recruitment and assessment

CSB subjects were recruited via Internet-based advertisements and therapist referrals. Healthy volunteers were recruited from community-based advertisements in East Anglia. Screening of the CSB participants was conducted using the Internet Sex Screening Test (ISST) [36] and an investigator-designed questionnaire. CSB subjects were interviewed by a psychiatrist to confirm they fulfilled diagnostic criteria for CSB (proposed diagnostic criteria for hypersexual disorder, criteria for sexual addiction [7], [37], [38]), focusing on compulsive use of online sexually explicit material.

All CSB subjects and age-matched healthy volunteers were male and heterosexual given the nature of the cues. Healthy volunteers were matched in a 2∶1 ratio with CSB subjects. Exclusionary criteria included being under 18 years of age, history of substance use disorders, current regular user of illicit substances (including cannabis), and having a serious psychiatric disorder, including current moderate-severe major depression (Beck Depression Inventory >20) or obsessive-compulsive disorder, or history of bipolar disorder or schizophrenia (Mini International Neuropsychiatric Inventory) [39]. Other impulsive/compulsive disorders or behavioural addictions (including problematic use of online gaming or social media, pathological gambling or compulsive shopping, childhood or adult attention deficit hyperactivity disorder, and binge-eating disorder) as assessed by a psychiatrist were exclusions.

Subjects completed the UPPS-P Impulsive Behaviour Scale [40], Beck Depression Inventory [41] and State Trait Anxiety Inventory [42] to assess impulsivity, depression and anxiety, respectively. The Obsessive-Compulsive Inventory-R assessed obsessive-compulsive features and the Alcohol-Use Disorders Identification Test (AUDIT) [43] assessed hazardous drinking behaviors. General Internet use was assessed using the Young's Internet Addiction Test (YIAT) [44] and the Compulsive Internet Use Scale (CIUS) [45]. The National Adult Reading Test [46] was used to obtain an index of IQ. Written informed consent was obtained, and the study was approved by the University of Cambridge Research Ethics Committee. Subjects were paid for their participation.

### Dot probe task

**Figure 1:**
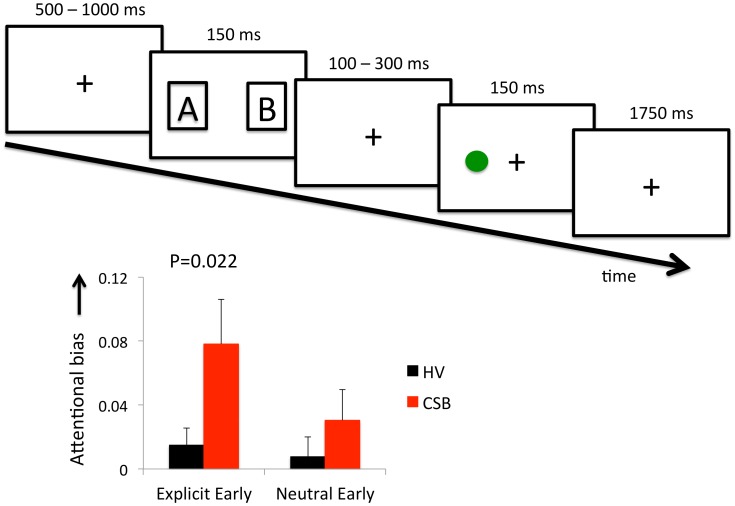
Dot probe task and attentional bias. Dot probe task. The cues (A, B) represent either a sexually explicit, erotic or neutral woman cue paired with a neutral furniture cue randomly presented on either side. Subjects are required to indicate the side in which the green target appears using one of two key presses. The graph represents attentional bias ((Reaction time (RT) for control – RT test cue)/(RT control + RT test cue)) for the early stimulus latency compared between subjects with compulsive sexual behavior (CSB) and healthy volunteers (HV). The error bars represent standard error of the mean.

Subjects viewed a computer screen while placing their left and right index fingers of the letter ‘s’ and ‘l’ of the keyboard. Subjects were told that they would see two images (including explicit images) followed by a green dot (Figure 1). The goal of the task was to indicate as quickly as possible the side in which the green dot occurred. Subjects were shown a central fixation cross (duration 500–1000 msec), followed by two images randomized to either the right and left of the fixation cross (duration 150 msec). The images disappeared followed by another central fixation cross (duration 100–300 msec), and the green target (150 msec). The green target appeared to the left or right of the screen in the center of where the images were previously shown. This was followed by another central fixation cross of 1750 msec to allow for the button response. The two images consisted of a cue and a neutral control image. There were 3 conditions: an Explicit cue (explicit images of consensual sexual interactions between a man and a woman), an Erotic cue (nude woman) and a Neutral person cue (dressed woman). In all cases these cues were paired with neutral Control images of furniture consisting pictures of single chairs. The task randomly cycled through the three conditions and through 15 different images from each of the condition categories. The task randomly cycled through thirty different neutral Control images of chairs. The green target randomly appeared on either side of the screen. Subjects underwent 5 practice trials followed by 40 trials per condition for a total of 120 trials. The task was coded using E-Prime 2.0 software.

The primary outcomes were the difference in reaction time (RTdiff) between the cues (erotic, explicit, neutral person) and paired neutral furniture cues ((RTneutral – RTcue)/(RTneutral+RTcue)) for the three conditions. As the latency of the stimulus prior to the target (stimulus onset asynchrony; SOA) has been shown to play a role in whether subjects engage in an early orienting response or a later inhibitory response [34], [35], the responses were divided into two separate categories based on stimulus latency (early SOA: 150 ms stimulus plus 100–200 ms fixation duration = 250–350 ms; late SOA: 150 ms stimulus plus 200–300 ms fixation duration = 350–450 ms).

### Statistical analysis

Subject characteristics and questionnaire scores were compared using independent t-tests or Chi-square tests. The RTdiff data were inspected for outliers (scores>3 SD above group mean) and tests for normality were conducted using Shapiro-Wilkes (P>0.05 was considered normally distributed). As the RTdiff scores for Explicit materials were not normally distributed (P = 0.007 for 250–300 msec; P = 0.04 for 350–450 msec), non-parametric analyses were conducted. We compared RTdiff between groups using Kruskal-Wallis test focusing on the early SOA. We focused on the *a priori* hypothesis that attentional bias to early SOA would be higher to Explicit versus neutral cues but not to a Neutral person versus neutral Control cue in CSB subjects compared to healthy volunteers. P<0.05 was considered significant. Other analyses such as Erotic versus neutral Control cues for early SOA and analyses for late SOA were conducted on an exploratory basis. To assess the influence of SOA, we also compared early versus late SOA for Explicit person cues using related-samples Kruskal-Wallis tests for each group on an exploratory basis.

## Results

**Figure 2:**
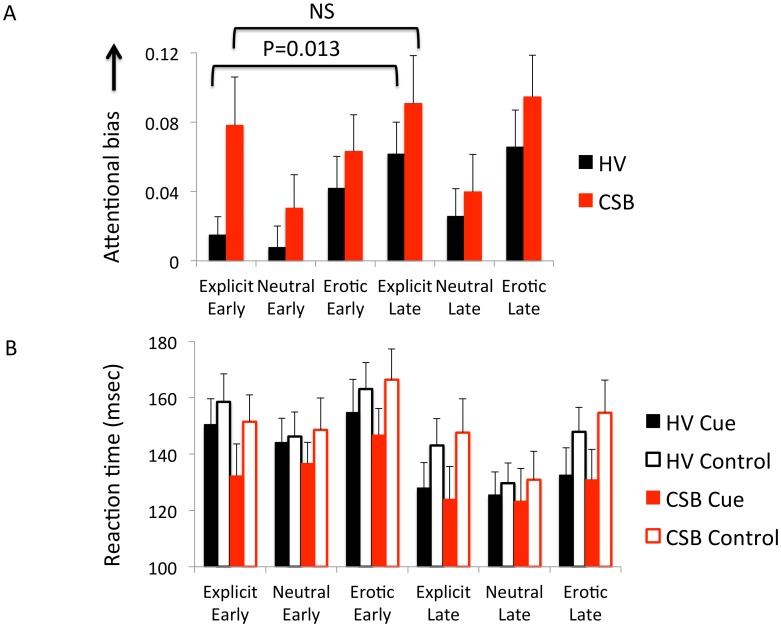
Stimulus latency and raw reaction time scores. A. Stimulus latency. The attentional bias score is shown for subjects with compulsive sexual behavior (CSB) and healthy volunteers (HV) as a function of stimulus latency (Early: 250–350 msec; Late 350–450 msec). B. Raw reaction time for cues and control stimuli for CSB and HV subjects. The error bars represent standard error of the mean.

Twenty-two heterosexual men with CSB (mean age 25.14 (SD 4.68) years) and 44 age-matched (mean age 24.16 (SD 5.14) years) heterosexual male healthy volunteers without CSB were assessed. Two of 22 CSB subjects were taking antidepressants or had comorbid generalized anxiety disorder and social phobia (N = 2) or social phobia (N = 1) or a childhood history of ADHD (N = 1). The characteristics of the CSB subjects are reported in Table 1. In the independent Kruskal-Wallis tests focusing on the *a priori* hypothesis, CSB subjects had greater attentional bias to Explicit stimuli (P = 0.022) but not to Neutral person cues (p = 0.495) for the early SOA (Figure 1). In exploratory analyses, there were no differences in attentional bias to Erotic stimuli (p = 0.529) for early SOA or to Explicit, Erotic or Neutral person cues for late SOA (p = 0.529, p = 0.382, p = 0.649) (Figure 2).

**Table 1:** Subject characteristics.

		CSB	HV	T/Chi square	P
Number		22	44		
Abstinence (days)		32(28.41)			
Education	High school	22	44	0.000	1.000
	Current Univ.	6	15	0.314	0.575
	College degree	3	6	0.000	1.000
	Univ. undergrad	9	15	0.295	0.587
	Masters degree	6	3	5.211	0.022
IQ		110.49(5.83)	111.98(8.71)	0.720	0.472
Relationship status	Single	10	18	0.124	0.725
	Curr. Relationship	7	17	0.295	0.587
	Married	5	9	0.045	0.831
Occupation	Student	7	16	0.133	0.715
	Part-time work	3	2	1.731	0.188
	Full-time work	12	23	0.030	0.862
	Unemployed	0	3	1.571	0.210
Medications	Antidepressants	2			
Body mass index		24.91(3.64)	23.1(4.29)	1.649	0.104
Binge Eating	BES	6.91(6.46)	5.83(6.58)	0.632	0.529
Alcohol use	AUDIT	7.13 (4.11)	6.81 (3.39)	0.337	0.738
Depression	BDI	11.03 (9.81)	5.29 (4.91)	3.184	0.002
Anxiety	SSAI	44.59(13.19)	36.27(13.83)	2.339	0.023
	STAI	49.54(13.91)	38.42(14.90)	2.920	0.005
Obsessive compulsive	OCI-R	19.23(17.38)	12.87(11.83)	1.753	0.084
Impulsivity	UPPS-P	150.83(17.95)	130.15(23.54)	3.622	<0.001

Abbreviations: CSB = subjects with compulsive sexual behavior; HV = healthy volunteers; BES = Binge Eating Scale; AUDIT = Alcohol Use Disorders Identification Test; BDI = Beck Depression Inventory; SSAI/STAI = Speilberger State and Trait Anxiety Inventory; OCI-R = Obsessive Compulsive Inventory; UPPS-P = UPPS Impulsive Behaviour Scale.

In exploratory analyses, healthy volunteers had greater attentional bias to Explicit stimuli in the late compared to early SOA (p = 0.013) but there were no differences between latencies in CSB subjects (p = 0.601). Similarly there were no differences between SOAs for the Neutral cue comparing early versus late SOAs for either the healthy volunteers (p = 0.404) or CSB subjects (p = 0.550). There were also no significant differences between groups for all raw RTs to the cues or neutral Control stimuli for all conditions and stimuli SOAs (all p>0.05) (Figure 2).

CSB subjects (attractiveness score: 8.16, SD 1.39) had similar ratings of attractiveness of the Neutral person cues relative to healthy volunteers (7.97, SD 1.31; p = 0.63). All subjects reported that they had not previously viewed the Explicit or Erotic stimuli.

## Discussion

Using the dot probe task, one commonly used to assess attentional bias in disorders of addiction, we show that CSB subjects have enhanced attentional bias towards sexually explicit stimuli but not to neutral cues.in early SOAs. These findings suggest a role for an early attentional orienting response underlying the relationship between CSB and sexually explicit cues.

The mechanisms underlying cue reactivity and attentional bias may reflect classical conditioning in which neutral stimuli (conditioned stimulus) are repeatedly paired with rewarding stimuli (unconditioned stimuli or sexual reward), such that the conditioned stimulus eventually elicits a conditioned response such as physiological arousal or craving. Following conditioning, these conditioned stimuli or drug cues acquire incentive-motivational properties thus acquiring salience, biasing attention and becoming ‘wanted’ [16], [17]. Further studies focusing on the role of conditioning in CSB subjects are indicated.

This predictive conditioned stimulus is believed to elicit an early orienting attentional response. Our task makes some attempt to address this initial fast automatic shifting of attention. Visual cues presented for less than 200 msec are more likely to reflect an initial attentional bias. Subjects require at least 50 msec to shift attention to a cue [47] and at least 150 msec to disengage from a simple cue towards another presented in a different spatial location [48]. In contrast, longer durations of 500 to 1000 msec may reflect multiple shifts of attention [49], reflecting disengagement and maintenance of attention, although not all studies have shown this [50]. In our study, the cue was presented for 150 msec followed by a fixation point for a total stimulus latency of 250 to 350 msec for the early SOA and 350 to 450 msec for the late SOA. We show that CSB subjects had greater attentional bias to the Explicit cue but not the Neutral cue compared to healthy volunteers for the early SOA but no group differences for the late SOA. We further show on an exploratory basis that healthy volunteers have an increase in attentional bias to the late relative to the early SOA. This suggests that the difference between groups in the early SOA may be related to enhanced early orienting mechanisms in the CSB group. The lack of difference between groups during the late stimulus latency is related to the enhanced attentional bias in healthy volunteers that may be temporally delayed and not representative of an early orienting response. Further studies designed to address earlier latencies of less than 100 to 200 msec are indicated. The role of abstinence may also have an effect on the duration of the visual cue. For instance, individuals in treatment for alcohol abuse were shown to have an attentional bias towards short duration alcohol cues (100 msec) but attentional avoidance with prolonged responding to long duration alcohol cues (500 msec) [34], [35]. Interpretation of findings from addiction Stroop tasks may be complicated by individuals' attempts to suppress or inhibit attentional bias or slowing of cognitive processes as a consequence of craving [26], [27]. These possible confounding factors may be less of an issue with the dot probe task, particularly with short SOAs, although in each task affected subjects are exposed to provocative stimuli that may induce arousal or craving. The SOA provides an index of the impact of the cue in visual perception and attention biases. Our preliminary study suggests that inhibitory processes may not relevant in CSB subjects at least for a latency of up to 450 msec. Future studies including longer duration cues of at least 500 msec are indicated to assess the potential roles for disengagement and maintenance of attention and inhibitory processes.

Alternatively, the results may represent the effects of familiarity with the category of Explicit stimuli in CSB subjects. A possible role for use-independent exposure has been suggested based on the lack of difference between attentional bias using a Stroop task in patients and a control group of employees in a substance use facility [51]. A recent study has also suggested a relationship between attentional bias in the maintenance phase in a visual search paradigm that correlates with use-independent exposure [52]. However, a study using the dot probe task that attempted to disambiguate familiarity from drug use studying sports enthusiasts versus non-sports enthusiasts failed to show any difference in attentional bias in early SOA for sports cues whereas a significant attentional bias was shown for active smokers in early SOA for smoking cues. This study which focused specifically on disentangling familiarity suggests that early capture of attentional bias in smokers as measured using the dot probe task is unlikely to be related to familiarity [53]. Thus, although familiarity with the stimulus category may play a role, it may be less likely to be relevant to the early capture of attentional bias in the dot probe task.

That the early orienting response to erotic stimuli was similar between CSB subjects and healthy volunteers was not unexpected, highlighting the salience of sexually relevant stimuli. Healthy male volunteers have shown enhanced initial orientation and maintenance of attention as measured by the number of first fixations and relative fixation time during eye-tracking to sexually preferred stimuli compared to non-preferred stimuli [54]. Similarly both healthy men and women focus longer on bodies than on faces of erotic stimuli [55]. Healthy males also have been shown to focus visual attention to women compared to men when viewing erotic and non-erotic stimuli [56]. Similarly, using the dot probe task with an SOA of 500 msec, enhanced attentional bias to sexual stimuli in healthy volunteers has been shown to correlate with higher sexual desire [57]. Thus, our findings suggest the explicit stimuli are differentially processed from erotic stimuli in CSB subjects and healthy volunteers. The explicit stimuli may be acting as conditioned cues similar to those in drug-cue-reactivity studies, hence provoking attentional facilitation and an early orienting response in individuals with CSB, whereas in healthy volunteers, the explicit stimuli may not act as conditioned cues but as sexually relevant stimuli, still provoking an eventual enhancement in attentional bias. In contrast, the erotic stimuli may be similarly processed in both groups as sexually relevant stimuli.

Our current findings dovetail with our recent observation that CSB subjects have enhanced activity to sexually explicit cues in the ventral striatum, amygdala and dorsal anterior cingulate activity, the same network activated in drug cue reactivity in disorders of addiction [58]. That this neural network correlates in CSB subjects with enhanced desire or wanting and not liking provides support for theories of incentive motivation being applicable to CSB. A quantitative meta-analysis of studies in cue reactivity across substances of misuse including alcohol, nicotine and cocaine showed overlapping activity to drug cues in the ventral striatum, dorsal anterior cingulate (dACC) and amygdala, with overlapping activity to self-reported cue-induced craving in dACC, pallidum and ventral striatum [59]. Using a modified dot probe task to assess attentional bias, alcohol dependent subjects were shown to have both an attentional bias towards the drug cues along with enhanced activity in the orbitofrontal cortex, ventral and dorsal striatum and amygdala [60]. The authors hypothesized that the extent of attention towards substance-related stimuli correlates with activity in reward-associated regions such as the ACC and striatum, due to cue-induced activation in these regions. Our current findings of enhanced attentional bias and an early orienting response to sexually explicit cues in CSB subjects lends further support to incentive salience mechanisms operating in CSB.

The study has multiple limitations. Only heterosexual male subjects were studied, and future studies should examine individuals of various sexual orientations and females [61]. Although the subjects fulfilled provisional diagnostic criteria and demonstrated functional impairment relating to sex using multiple validated scales, there currently exist no formal diagnostic criteria for CSB, thus limiting generalizability of the findings. Future studies should examine whether these measures may be state or trait related. The restricted age range may also limit generalizability. As fewer different neutral Control images were randomly shown relative to the different cue images, the informative value of the neutral Control images would be less than the cue images as they were presented less frequently. The design is similarly biased towards the cue pictures given that the cues are people as compared to objects. Future designs should match the frequency of image presentation for the cue and control stimuli and match for categories of people rather than objects (e.g., two people interacting as a match for the Explicit condition).

That attentional bias is a feature across drug and natural rewards suggests a potential role for attentional bias as an important construct in the dimensional approach towards disorders [62]. Our findings of enhanced attentional bias in CSB subjects suggest possible overlaps with enhanced attentional bias observed in studies of drug cues in disorders of addictions. These findings converge with recent findings of neural reactivity to sexually explicit cues in CSB in a network similar to that implicated in drug-cue-reactivity studies and provide support for incentive motivation theories of addiction underlying the aberrant response to sexual cues in CSB.
